# Bayesian Belief Network-based assessment of nutrient regulating ecosystem services in Northern Germany

**DOI:** 10.1371/journal.pone.0216053

**Published:** 2019-04-30

**Authors:** Sabine Bicking, Benjamin Burkhard, Marion Kruse, Felix Müller

**Affiliations:** 1 Institute for Natural Resource Conservation, Department of Ecosystem Management, Kiel University, Kiel, Germany; 2 Institute of Physical Geography and Landscape Ecology, Leibniz Universität Hannover, Hannover, Germany; 3 Leibniz Centre for Agricultural Landscape Research ZALF, Müncheberg, Germany; National Institute of Geophysics, Geodesy and Geography, BULGARIA

## Abstract

This study aims to assess the potential supply of the ecosystem service (ES) nutrient regulation on two spatial scales, the federal German state of Schleswig-Holstein (regional) and the Bornhöved Lakes District (local), exemplarily for the nutrient nitrogen. The methodology was developed using the ES matrix approach, which can be applied to evaluate and map ES at different geospatial units such as land use/land cover classes. A Bayesian Belief Network (BBN) was constructed in order to include additional spatial information on environmental characteristics in the assessment. The integration of additional data, which describes site-specific characteristics such as soil type and slope, resulted in shifted probability distributions for the nutrient regulation ES potential. The focal objective of the study was of methodological nature: to test the application of a BBN as an integrative modelling approach combining the information from the ES matrix with additional data sets. In the process, both study areas were assessed with a regional differentiation with regard to the predominant landscape types. For both study areas, regional differences could be detected. Furthermore, the results indicate a spatial mismatch between ES demand and supply of the nutrient regulation potential. Land management and agricultural practices seem not to be in harmony with the spatial patterns of the environmental characteristics in the study areas. The assessment on the local scale, which comprised higher resolution input data, emphasized these circumstances even more clearly.

## Introduction

Research on the interrelations between human activities and the environment is of key importance for our society. Increased understanding of the environment and of the effects of our behaviour on the environment will support sustainable policy and decision making. In particular, decision making with regard to spatial planning, land and resources management and agricultural practices has to be founded on precise information in order to support sustainable land use. In this regard, the concept of ecosystem services (ES) is a valid assessment approach. The concept commonly differentiates between three ES categories: regulating, provisioning and cultural ES, all of which contribute to human well-being (i.e. [1–6]) ES analyses can be used to support spatial planning and attaining sustainable land management [7,8]. The relevance of this approach is consolidated also in European policy. Through the Biodiversity Strategy 2020, the European Union called upon its member states to map and assess the states of their ecosystems and the services they provide (Target 2 Action 5 of the Biodiversity Strategy; see [9]). This demand emphasizes the need for simple and applicable methods for ES assessment and mapping. In several previous studies, the assessment and mapping of the ES supply and demand has been based on the ES matrix approach by Burkhard et al. ([2,10] also in [11–13]). The ES matrix has been used to distinguish, amongst others, ES supply for different land cover types. CORINE land cover data [14] from the European Union has often been selected as underlying data set [10,15].

When using land use land cover (LULC) data such as CORINE, the spatial ES modelling is dependent on the prevailing land cover types, which usually are strongly related to vegetation and land use patterns. Based on that, ES can be qualitatively mapped based on expert knowledge and on the matrix values provided by Burkhard et al. [10] related to the CORINE land cover classes. The previous ES assessment based upon the ES matrix approach by Burkhard et al. [2,10,16] aimed to assess potential ES supply based on the CORINE land cover classes. The land cover classes were used as proxies for ES potentials based on simple causal relationships such as food supplied by agricultural land uses, timber by forests and freshwater by rivers or lakes. Additionally, Burkhard et al. [2,10,16] included normalized quantitative as well as qualitative data into the ES matrix in order to come up with a valuation of ES potentials for the land cover classes on a relative scale from zero to five.

This so called ‘tier 1’ ES mapping approach [17] has been proven to be efficient to raise awareness and for gaining an overview of ES in study areas [2,15,18,19]. However, it is questionable whether ES allocation solely based on one spatially explicit data set (such as land cover) is sufficient in order to represent local ecosystem conditions [10,15]. As most study areas are not homogenous, but differ, e.g., in geomorphology, soil type and texture, it is reasonable to suppose that ES supply differs spatially throughout the study area, for instance also within one land use class [20].

This study therefore aims at assessing the influence of several site-specific characteristics and properties on the potential supply of a selected ES. The research was executed for the ES nutrient regulation, which is defined as the ability of an ecosystem to recycle nutrients [10]. We refer here to ES potential, which has been defined by Burkhard et al. [10] as the hypothetical maximum yield of selected ES. ES potential is different from ES flow, which describes ES that are actually used in a specific area and time, driven by demand for ES [21].

The assessment was tested on two spatial scales: regional and local. The northern German federal state of Schleswig-Holstein (regional scale) and the Bornhöved Lakes District (local scale) were selected as study areas. A Bayesian Belief Network (BBN) was developed for the analysis. BBNs are multivariate statistical models which feature a probabilistic modelling approach [22,23]. Casually speaking, BBNs represent causal graphs, including arcs connecting variables. The arcs depict the direct causal influences of the variables they connect [23]. The conclusions one can draw from a BBN are probabilistic [24]. BBNs have only recently been introduced into ecological modelling [25]. A few studies have applied BBNs in the context of ES assessments (e.g. [5,24,25]), mainly using landscape structures to predict the probability of ES supply [22,26–28]. Thereby one focal advantage arises from the possibility to integrate data and knowledge from different fields and origins and to link quantitative and qualitative information. These applications seemed promising and roused our interest to apply the approach in ES research and in our study.

The BBN was constructed based on the ES matrix approach by Burkhard et al. [10]. The goal of the research was to come up with a more differentiated ES classification for the nutrient regulation ES potential. The approach integrated additional data on site-specific social-ecological system properties with the original nutrient regulation ES potential based upon the matrix approach which uses CORINE LULC as proxy for the ES potential. Thus, the BBN has been set up to combine further relevant environmental data sets, which describe the spatial distribution of significant site-specific characteristics, such as soil texture and slope and the results of the ES matrix approach by Burkhard et al. [10] into one assessment. By using BBN approach, it is possible to incorporate regional spatial differentiations within the study areas into the ES assessment and increase the study’s comprehensiveness.

The study can be understood as an attempt to test the convenience of integrating various data sets and information by means of a BBN in the two study areas. The aim was not to compile a model that describes reality in a complete and all-embracing manner. Instead we wanted to test the influence of integrating additional data in the evaluation of the nutrient regulation ES potential. Special attention was paid to the assessment of regional differences within the two study areas which were examined based on the prevailing landscape types. The BBN was used in both study areas to examine the assumption that the site-specific characteristics of the prevailing landscape types strongly influence the nutrient regulation ES potential. Besides, the issue of spatial scale was explored with regard to data mining, implementation and running of the BBN and interpretation of the results.

Resulting from the issues described above, the following hypotheses were tested in this study:

1. The inclusion of data on site-specific properties for the assessment of the nutrient regulation ES potential results in a more scattered distribution of the ES potentials compared to the ES matrix values provided by Burkhard et al. [10].
2. The assessment of the nutrient regulation ES potential results in a regional differentiation in Schleswig-Holstein.
3. The probability distribution of the nutrient regulation ES potential in the Bornhöved Lakes District resembles the distribution for Schleswig-Holstein.

## 2 Background information

This section comprises information on the study areas followed by some general knowledge about nutrients focusing on nitrogen and the ES nutrient regulation.

### 2.1 Physical features of the study areas

In the following two paragraphs, the two study areas are described and their characteristics are depicted.

#### 2.1.1 Schleswig-Holstein

**Fig 1:**
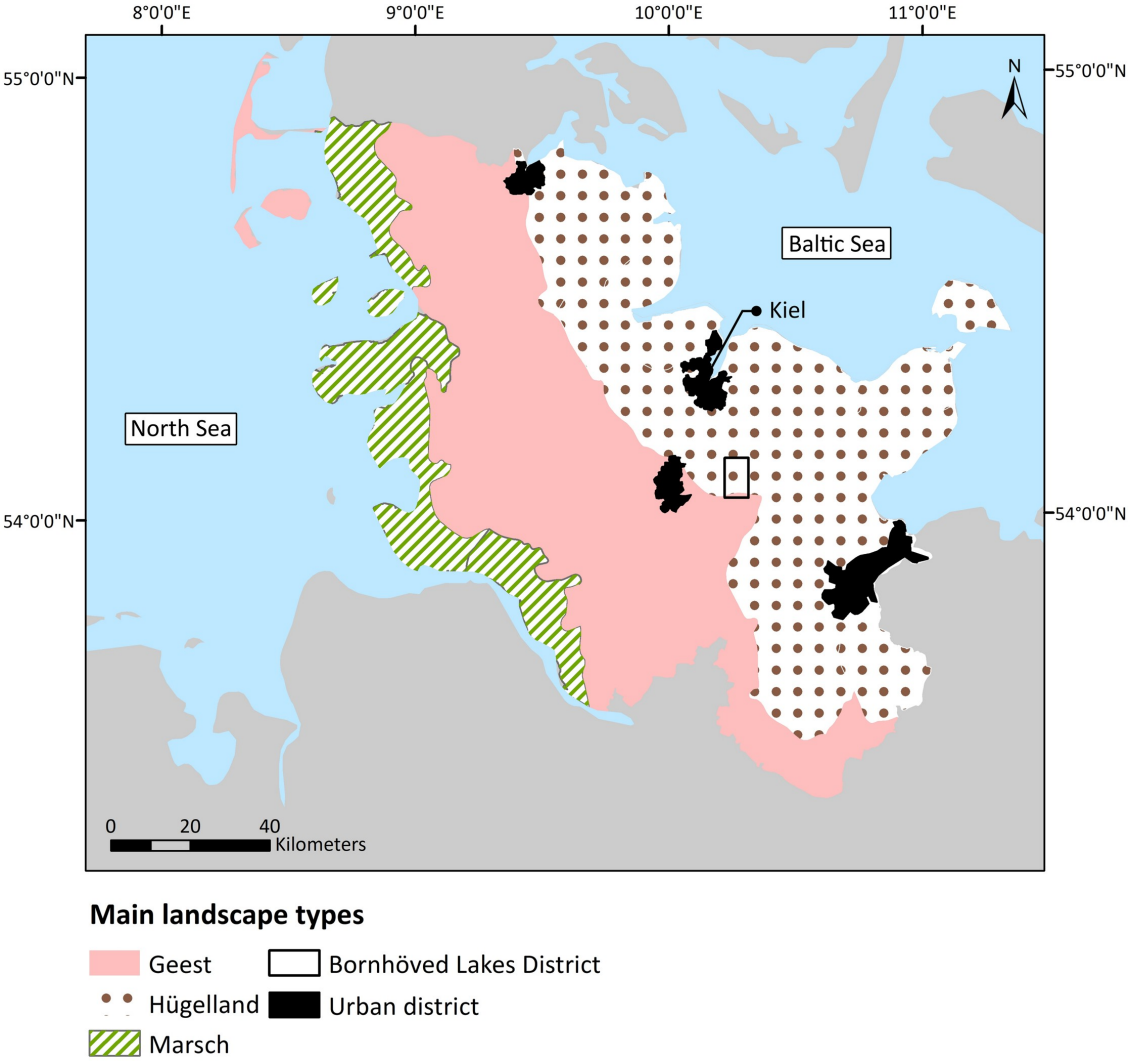
Schleswig-Holstein study area, showing the differentiation between main landscape types and the location of the local case study Bornhöved Lakes District (based on data from Landesamt für Landwirtschaft, Umwelt und ländliche Räume and Natural Earth).

The federal state of Schleswig-Holstein is located in Northern Germany and has a spatial extent of 15’802 km^2^ [29]. The adjacency of the North Sea to the West and the Baltic Sea to the East of the study area (Fig 1) leads to maritime and humid climatic conditions, with an annual mean temperature of around 8°C and a precipitation average of approximately 840 mm [30]. The Pleistocene, the last glacial period, played an important role in the creation and formation of today’s landscapes. Especially the last two glaciations of the Pleistocene, the Saalian and the Weichselian glaciation, highly influenced the geological and geomorphological conditions of Schleswig-Holstein [31]. Particularly, the varying expansions of the glaciers during the Saalian and the Weichselian glaciation led to the regional natural conditions within the study area [31,32]. As a consequence, the landscapes of Schleswig-Holstein reveal three main landscape types [32,33]: Hügelland, Geest and Marsch (Fig 1).

Contrary to the Saalian glacier, the Weichselian glacier only covered the Eastern part of Schleswig-Holstein. During that time, the area of the Geest embodied the outwash plains of the glacier [31,33]. Therefore, the area of the Geest is characterized by poorer, sandy soils [31,33]. Due to erosion, the landscape of the Geest features only little relief. The landscape of the Hügelland demonstrates the impact of the Weichselian glaciation on the area, as rolling hills formed by the moraines and small lakes as well as deep embayments prevail [31–33]. Resulting from the geological history, the soils of the Hügelland are rather fertile. The area of the Marsch, in the Western part of Schleswig-Holstein, is also characterized by fertile soils. Unlike the Geest and Hügelland, the Marsch originates from Holocene marine sediments and is thus the youngest of the three landscape types [32,34].

The diversity of the varying landscape types within the study area is of great relevance for this research, because the influence of site-specific properties, originating from these differences, on the nutrient regulation ES potential was assessed. The distribution of several site-specific characteristics, e.g. soil types, correlates with these main landscape types [35].

#### 2.1.2 Bornhöved Lakes District

The study area representing the local scale is called Bornhöved Lakes District. It is located within the federal state of Schleswig-Holstein, more precisely around 30 kilometres south of the state capital city Kiel. The area has been selected as case study area for several previous research and monitoring projects [4,36,37]. The case study area that was selected for ES research has an extent of 60 km^2^ [4,38]. The Northern and the central parts of the study area belong to the Hügelland, the Southern part to the Geest (Fig 1). Thus, great influences of the Weichselian glaciation can be seen in the landscape [37]. There are six glacially-formed lakes surrounded by forests and agricultural areas in the Bornhöved Lakes District, which has been characterized by Fränzle et al. [37] and Fohrer and Schmalz [39] as a representative landscape for Northern Germany.

### 2.2 Ecochemical features of the investigated nutrients

Nutrients are chemical elements that plants and animals require for growth, reproduction and survival [40,41]. On Earth, there are constant and natural cycles of nutrients [42,43]. The cycles depicts how nutrients enter the ecosystem, how they are transferred within the ecosystem and how they eventually leave the ecosystem. Nutrients can be divided into two categories: mineral and non-mineral elements [44]. Non-mineral elements, which are used in large quantities by all organisms, are carbon, hydrogen and oxygen. However, also mineral elements are indispensable for life to exist. For agroecosystems and plant growth in general, the following nutrients are of great relevance: nitrogen, phosphorus, potassium, calcium, magnesium and sulphur [45]. Nutrients move within an ecosystem through the biosphere, hydrosphere, lithosphere and atmosphere [46]. Plants take up nutrients from the soil. Subsequently, the plants are consumed by animals or human beings. After physical ingestion processes, the nutrients are excreted. Otherwise they return to the environment as soon as the organism dies. The organic matter is broken down in the soil by microorganisms which transform the nutrients back to their original mineral form [46]. However, this cycle varies for the different nutrients because of their biogeochemical properties.

#### 2.2.1 Nutrient regulation

Nutrient regulation has been defined by Burkhard et al. [10] as the ability of an ecosystem to recycle nutrients. Other studies, which focused on the ES provided by soils, referred to filtering, absorption and retention of nutrients in this context [47–49]. The ES nutrient regulation is highly relevant for agricultural practices and land management. Under natural conditions, a steady-state is reached with regard to the nutrient pool [43]. This means, that in- and outputs are in balance [43,50,51] and the cycle is almost closed [44].

Agricultural practices generate vast changes in the natural nutrient cycles [52,53]. The usage of fertilizers and the demand for high yields result in an artificial opening of the nutrient cycle [43,44]. As a consequence of the opened nutrient cycle, affected areas may suffer from nutrient deficiency or nutrient oversupply. Both of these circumstances degrade the environment and jeopardize biodiversity and human health. The nutrient regulation ES combats both of these issues, ensuring a functioning and sustainable nutrient cycle [43]. Nutrient regulation varies for different ecosystems. Especially natural ecosystems such as forests and grasslands have a high potential for nutrient regulation [10,20]. Besides LULC, other factors are of relevance for determining the nutrient regulation ES potential, such as slope [20], soil conditions and climatic conditions. As summarized by Bicking et al. [15], the beneficiaries of the ES nutrient regulation are diverse. On the one hand, the society as such strives for a clean environment [48,54]. In consideration of the quantity of the associated directives and regulations arising from national as well as European legislation, also politics as such can be defined as a beneficiary. On the other hand, according to Power [55], agriculture provides and consumes ES at the same time. Amongst others, agriculture provides crops for fodder and food production. In order to ensure this provision of ES, the agricultural system is dependent on other ES, such as nutrient regulation [2,55].

The biogeochemical properties of different nutrients vary and thus associated processes in the environment differ. For reasons of simplification, we analysed the nutrient regulation ES potential exemplarily for the element nitrogen alone.

#### 2.2.2 Nitrogen

**Fig 2:**
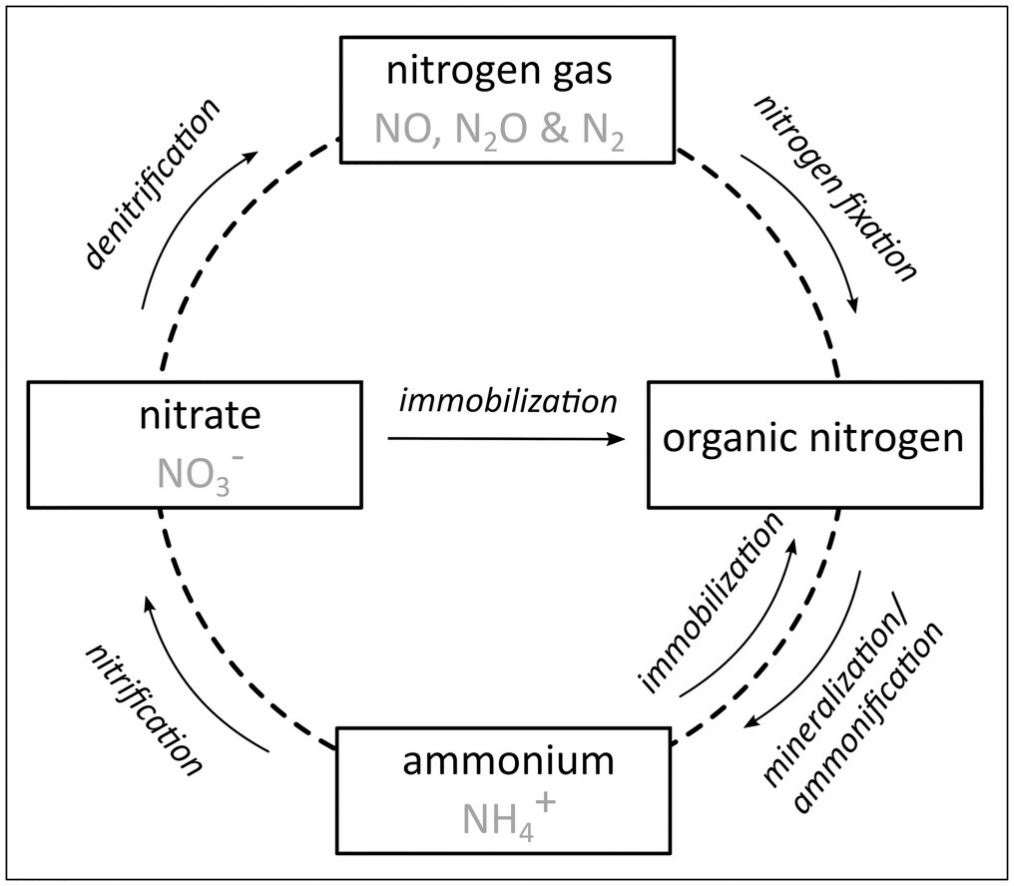
Simplified nitrogen cycle (after [64]).

Nitrogen occurs naturally in inert as well as in reactive forms on our planet [56]. The elementary nitrogen which makes up approximately 78% of our atmosphere is the inert nitrogen gas (dinitrogen, N_2_) [44,57]. Due to natural processes as well as anthropogenic activities, reactive forms of nitrogen are created from the inert nitrogen gas [44,58]. The natural processes are biological nitrogen fixation and lightning [44]. The anthropogenic activities which create reactive nitrogen comprise fossil fuel combustion and the creation of synthetic fertilizer through the Haber-Bosch process [44,57]. Reactive forms of nitrogen are [59]: nitrate (NO_3_^-^), ammonia (NH_3_), ammonium (NH_4_^+^), nitric oxide (NO), nitrous oxide (N_2_O) and organic bound nitrogen (N_org_). All organisms are dependent on reactive nitrogen as a building component for proteins and their hereditary materials [58]. Plants are dependent on that essential nutrient for growth, reproduction and survival. The nitrogen cycle is influenced by biological processes and varies according to climatic conditions and depends on soil properties (both physical and chemical) [46,53]. There are several sources for nitrogen inputs into an ecosystem, including biological fixation, atmospheric fixation (lightning) and nitrogen deposition, industrial fixation (mineral fertilizer), from soil organic matter, crop residues and animal manures [60,61]. Generally speaking, nitrogen is available to plants as ammonium or nitrate [53,60,62]. Other nitrogen sources must be converted before being taken up by plants. Nitrogen in soils undergoes several transformations [53,60,63,64] (Fig 2):

1. Nitrogen fixation: The conversion of atmospheric nitrogen to a plant available form.
2. Mineralization: Organic nitrogen in the soil is converted into inorganic nitrogen (ammonium) by microbial activity.
3. Nitrification: The biological transformation from ammonium to nitrate.
4. Denitrification: Bacterial transformation from nitrate to gaseous nitrogen which is transferred to the atmosphere.
5. Immobilization: The conversion of inorganic nitrogen to organic nitrogen by i.e. micro-organisms.

Alongside biological transformations, physical processes, such as leaching, are also relevant for the nitrogen cycle. Leaching describes the downward movement of soluble nitrate in the soil with soil water as transport medium [47,60]. Leached nitrate enters either the ground or surface water and degrades the quality of water bodies through eutrophication [20,47–49,65]. Thus, eutrophication results from the excess input of nitrate or other nutrients into a water body [66]. All in all, leaching, denitrification, volatilization, crop removal and soil erosion and runoff can possibly lead to nitrogen losses in a soil system. Sustainable management of nitrogen is of great importance, as insufficient nitrogen availability in our soils decrease crop production and excess quantities pollute our environment. When excess nutrients are introduced to the ground or surface water, eutrophication processes occur and endanger the ecological status of our water bodies.

## 3 Materials and methods

### 3.1 Bayesian Belief Networks

Bayesian Belief Networks (BBNs) are multivariate statistical models. They feature a probabilistic modelling approach [22–24] and are characterized by a high model transparency [22]. The model as such consists of two parts: a direct acyclic graph (DAG) and conditional probability tables (CPTs) [22,23,67,68]. The DAG depicts the dependencies between the different variables which are included in the BBN. The DAG illustrates the structure of the probabilistic domain [23]. The CPTs on the other side store the strength of the links in the graph [69]. Within the DAG, dependencies are depicted as arrows representing cause-effect relations between the variables or more precisely so-called nodes [22,23]. Within the acyclic graph, arrows lead from parent nodes to child nodes [22,67,70,71]. The development of the DAG can be based on different techniques, e.g. system understanding by experts or learning from empirical observations [22,71]. All variables which are included in the BBN contain a limited number of states. Their realized value must belong to one of these states [22]. The BBN features the ability to consider uncertainties as the realized value of a particular variable can be allocated to multiple states using probabilistic methods. The CPTs store the conditional probabilities indicating the strengths of the causal relations between the different nodes [22]. The BBN can be used as an integrated modelling framework bridging the gap between quantitative and qualitative data [22,68,70,72]. To obtain quantitative model outputs, Bayesian inference is used to propagate these probabilities through the network [69,71]. Bayesian inference is based upon the Bayesian theorem [73,74]:

$$\text{Pr}\;\left(A|B\right)\;=\;\frac{\text{Pr}\;\left(B|A\right)\text{Pr}\;(A)}{Pr\;(B)}$$ 1

The formula indicates that the conditional probability, or posterior probability, of an event A after event B (Pr(*A*|*B*)) is observed in terms of the prior probability of A (Pr(*A*)), the conditional probability of B given A (Pr(*B*|*A*)) and the prior or marginal probability of B (Pr(*B*)), which acts as a normalizing constant. In other words, the BBN is composed of a set of interconnected nodes. Each node represents one variable within the model. For each variable/node, there are different possible states. The causal relationship between the variables is depicted in the form of arcs. The probability of the individual states of the nodes is determined from the probability of each possible state of all connected nodes and their causal relationship.

In summary, BBNs represent causal graphs and each arc within the graphs represents a direct causal influence between the nodes that it connects [23]. The structure and the numerical probabilities can be derived from diverse sources, among which measurements, objective frequency data, expert evaluation or a mixture of these sources [23] can play an important role.

Landuyt et al. [5] applied the BBN approach in 2012 in order to predict multiple ESs delivered by a catchment area. They included different scenarios in order to evaluate alternative ecosystem management practices. Information from existing models, literature and expert evaluation was used to set up the BBN. In 2014, Landuyt et al. [27] developed a BBN to assess the soil organic carbon (SOC) storage as an indicator for the ES global climate regulation. The BBN was based upon data from an empirical study on SOC storage. The DAG was structured, and the CPTs filled according to the results of the empirical study. Additional information was derived by Landuyt et al. [27] from further literature studies. Barton et al. [26] used a BBN to assess preferred combinations of trees in live fences and on pastures in silvopastoral systems and the corresponding provision of ESs. They developed the BBN based upon local farmer knowledge, the farmers’ expressed needs and aspirations, and scientific knowledge from literature analysis and fieldworks.

For this study, we used the Software GeNIe Modeler developed by BayesFusion LLC. GeNIe is a graphical user interface to SMILE (Structural Modeling, Inference, and Learning Engine). The software allows for interactive model building and learning [23].

#### 3.1.1 Structuring the BBN and finding cause-effect relations

We built the DAG by aligning our knowledge of the analysed social-ecological system to the data available for this research. The model development was based on the protocol by Cain [75]. In the first step, we developed a general network structure including three nodes:

1. the predominant landscape types,
2. the preliminary nutrient regulation ES potential (based upon the ES matrix approach), and
3. the new nutrient regulation ES potential.

In general, the DAG of the BBN was developed based upon system understanding by an interdisciplinary group of experts. The group of experts consisted out of ten experts from different research domains, e.g. agricultural sciences, forestry, soil sciences, (physical) geography and biology. Further variables (nodes), representing environmental characteristics, and connections were added to the network which seemed relevant according to various literature sources and our general system understanding. The preliminary DAG was evaluated by the group of experts in a roundtable discussion where several nodes and dependencies have been adjusted according to the general census. Thus, the DAG was constructed based on literature knowledge, regional information and on the experts’ understanding of the causal relations between the relevant variables in the model.

In addition to the nodes aiming to describe the nutrient regulation ES potential, further nodes/variables were added, as another aim of this study was to assess ES budgets based on ES supply and demand balances. Therefore the following variables were added to the network: nutrient regulation ES demand, reclassified nutrient regulation ES potential and nutrient regulation ES budget. The ES demand has been defined by Jones et al. [76] as the quantity of beneficiaries and their ES needs. As described in Chapter 2.2.1, the beneficiaries of the nutrient regulation ES are diverse. For this modelling exercise, the nutrient surplus (exemplarily for nitrogen) which has been calculated as indicator for the nutrient regulation ES demand by Bicking et al. [15], was added to the BBN.

Bicking et al. [15] investigated the nutrient regulation ES demand in Schleswig-Holstein. They defined the nutrient regulation ES demand as the nutrient surplus and performed the ES assessment for the nutrient nitrogen. In order to obtain data on the nutrient surplus, they calculated nutrient budgets, subtracting nutrient outputs from nutrient inputs. The following parameters were included in the calculation [15]: organic fertiliser (from livestock) and mineral fertiliser with corresponding losses, compost, biological fixation, atmospheric nitrogen deposition, yield, digestate from biogas plants and sewage sludge. Bicking et al. [15] performed the calculation on two different spatial scales. For the whole area of Schleswig-Holstein (regional scale), the ES demand was calculated on the level of municipalities and in the area of the Bornhöved Lakes District (local scale), the ES demand was calculated for the corresponding CORINE LULC polygons.

The ES budget assessment, aiming to provide information on sustainable or unsustainable conditions, was included as a very rough estimation in order to give an idea of areas of nutrient regulation ES over, respective undersupply. In the next step, we incorporated the available data sets (see Chapter 3.2 below) and assessed the correlation directions (positive or negative) and degrees (strengths) where possible in order to derive the CPTs. In these cases, the numerical probabilities were completely derived from the input data sets using the automatic learning function of the BBN software (learn parameters of an existing network). However, several cause-effect relations of our BBN (CPTs) remained open and could not be determined statistically based on the input data sets. In that case, literature evaluation in combination with expert knowledge was harnessed. The above-mentioned group of experts was consulted. The expert evaluation was executed in several rounds of valuation and all results were reviewed repeatedly. The format of the expert evaluation rounds were roundtable discussions and the debates were conducted openly in several rounds until a general approval was obtained concerning the degree of correlation (0–1) between the corresponding nodes. The results were filled into the respective CPTs.

**Fig 3:**
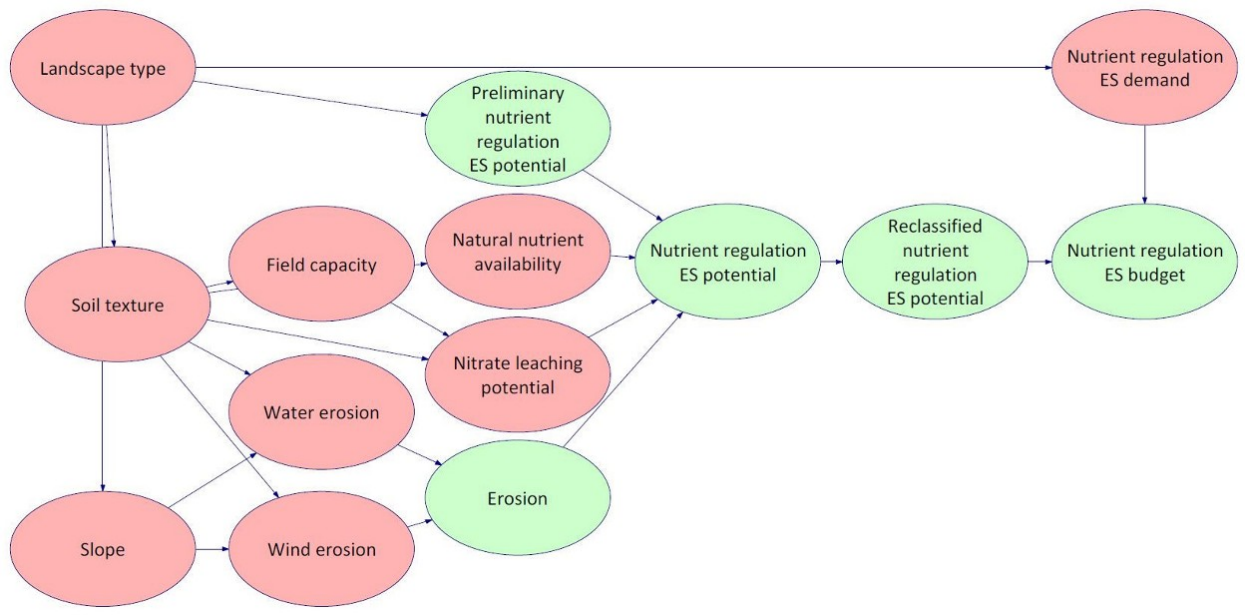
Structure of the BBN. Colours are indicating quantitative (red) /qualitative approach (green).

Fig 3 indicates which nodes, more precisely the CPTs of the nodes, are based on quantitative and qualitative approaches.

The model consists in total of 14 nodes (Fig 3). The variables can fall into different states for each node. Table 1 gives an overview on the possible states for each of the nodes. The number of states for each node is dependent on the available data and the general assertion of the respective node. If possible and reasonable, we tried to define three states for each node. Furthermore, we tried to conform to classifications and evaluations of the original input data sets (see Chapter 3.2). An exception is the definition of the states of the node slope, where we used a quantile classification.

**Table 1:** Nodes and corresponding states.

Node	States	Description
Landscape type	Hügelland	Landscape region: Hügelland
	Geest	Landscape region: Geest
	Marsch	Landscape region: Marsch
Soil texture	sand	Predominant soil texture: sand
	peat	Predominant soil texture: peat
	silt_clay	Predominant soil texture: silt/clay
	other	Predominant soil texture: other
Slope	low	Slope: 0–0.2039°
	medium	Slope: 0.2039–0.6581°
	high	Slope: 0.6581–13.4431°
Field capacity	low	Field capacity: < 200 mm
	medium	Field capacity: 200–300 mm
	high	Field capacity: > 300 mm
Wind erosion	no	No wind erosion
	low	Low wind erosion
	medium	Medium wind erosion
	high	High wind erosion
Water erosion	no	No water erosion
	low	Low water erosion
	medium	Medium water erosion
	high	High water erosion
Natural nutrient availability	low	< 300 kmol_c_/ha
	medium	300–600 kmol_c_/ha
	high	> 600 kmol_c_/ha
Nitrate leaching potential	low	Low nitrate leaching potential
	medium	Medium nitrate leaching potential
	high	High nitrate leaching potential
Erosion	low	Low erosion potential
	medium	Medium erosion potential
	high	High erosion potential
Preliminary nutrient regulation ES potential	P_no	No relevant potential
	P_1	Low relevant potential
	P_2	Relevant potential
	P_3	Medium relevant potential
	P_4	High relevant potential
	P_5	Very high relevant potential
Nutrient regulation ES potential	P_no	No relevant potential
	P_1	Low relevant potential
	P_2	Relevant potential
	P_3	Medium relevant potential
	P_4	High relevant potential
	P_5	Very high relevant potential
Reclassified nutrient regulation ES potential	low	Low potential supply of nutrient regulation
	medium	Medium potential supply of nutrient regulation
	high	High potential supply of nutrient regulation
Nutrient regulation ES demand	low	Nitrogen surplus: < = 40 kg N/ha
	medium	Nitrogen surplus: 41–60 kg N/ha
	high	Nitrogen surplus: > 60 kg N/ha
Nutrient regulation ES budget	sustainable	Potential higher than demand for nutrient regulation ES
	unsustainable	Potential lower than demand for nutrient regulation ES

#### 3.1.2 Validation of the BBN

**Fig 4:**
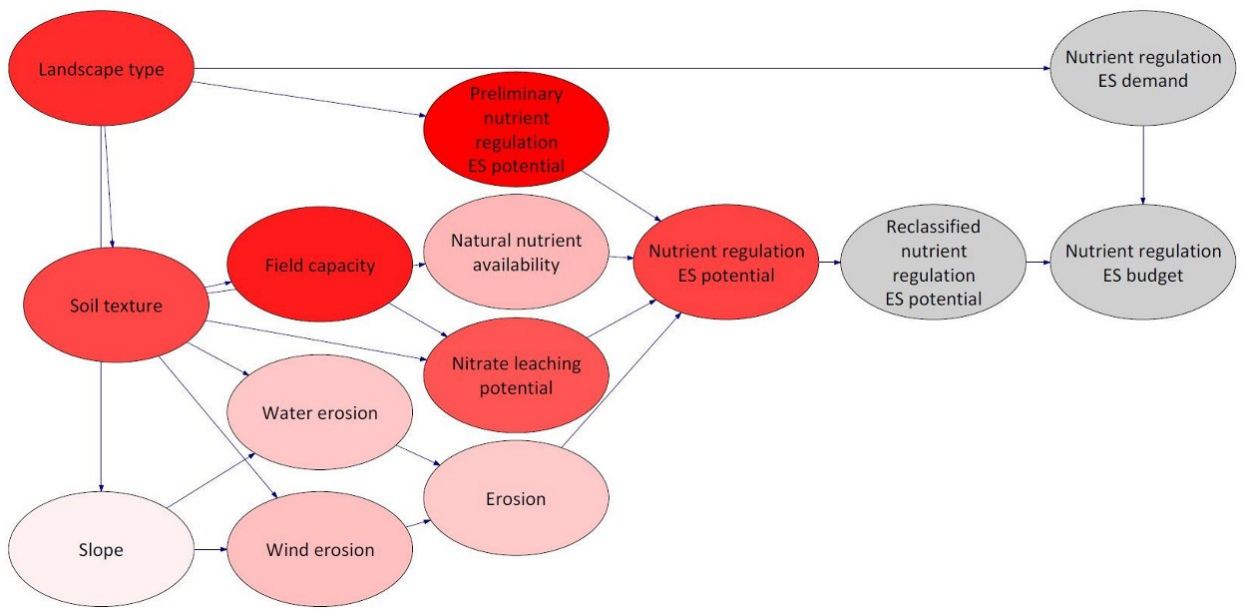
Sensitivity analysis for the node nutrient regulation ES potential in Schleswig-Holstein. Intensity/strength of red colour indicates strength of influence.

For model validation, we chose different types of validation processes: expert-based, sensitivity analysis and k-fold cross validation. In a first step, in order to validate the structure and validity of the model, experts were consulted. Concerns were related to oversimplification of the model structure and subjectivity which comes along with the generation of the CPT table. In a next step, a sensitivity analysis was executed for the target variable nutrient regulation ES potential (Fig 4). Such a sensitivity analysis determines the influence of the other variables on the target variable [23,71], indicating the effect of minor changes on the probability of a state on the probability distribution of the target variable [69]. Thus, these variables affect the results more significantly. Within the BBN software GeNIe, an algorithm by Kjaerulff and van der Gaag [77] is implemented which performs the sensitivity analysis. The algorithm calculates for one or more target nodes, derivatives of the posterior probability distributions over the target nodes over each of the numerical parameters. The higher the derivative for a variable, the larger is the influence on the respective posteriors [23]. The sensitivity analysis (Fig 4) reveals that the target variable, which is the nutrient regulation ES potential, is most sensitive to the variables landscape type, soil texture, field capacity, nitrate leaching potential and preliminary nutrient regulation ES potential.

When learning and evaluating a model with the same data set, cross-validation is an appropriate evaluation method [23]. Cross-validation divides the data set into subsets for training and testing. The *k*-fold cross-validation, which is included in GeNIe, divides the data set into *k*-parts. Each data set is of equal size. *K* − 1 subsets are used for training and last *k*th subset is used for testing. This procedure is repeated *k* times [23]. The performance measure of the model is the average of all individual performances [69]. Thus, the whole data set can and has been used for training and testing [69].

### 3.2 Input data for the BBN

The quality of the input data is key for the applicability of BBN-based assessments. Data sets for the two study areas were collected in the form of digital maps (ArcGIS shape files). The data were extracted from the shape files using a pre-defined grid in the ArcMap 10.3 GIS software. The grid size was approximately 200 m, resulting in more than 300’000 data points for the study area of Schleswig-Holstein and around 1’500 data points for the Bornhöved Lakes District area. Table 2 gives an overview of the data sets which were used for this research in both study areas. The data sets have been cleaned, processed and discretized in order to fulfil the input requirements of the BBN. All relevant information has been combined in one data set for each of the two BBNs—for Schleswig-Holstein and for the Bornhöved Lakes District.

**Table 2:** Input data.

Data set	Description	Resolution/ Scale	Source
CORINE LULC	Vector	10 ha	Bundesamt für Kartographie und Geodäsie (BKG, eng.: Federal Agency for Cartography and Geodesy) (http://www.geodatenzentrum.de/); European Environment Agency/ Copernicus (https://land.copernicus.eu/pan-european/corine-land-cover/clc-2012/)
Nitrate leaching potential	Vector	1:250 000	Landesamt für Landwirtschaft, Umwelt und ländliche Räume (LLUR, eng.: State Agency for Agriculture, the Environment and Rural Areas) (http://www.umweltdaten.landsh.de)
Nutrient availability in the effective root zone	Vector	1:250 000	LLUR (http://www.umweltdaten.landsh.de)
Field capacity in the effective root zone	Vector	1:250 000	LLUR (http://www.umweltdaten.landsh.de)
DEM	Raster	200 m	BKG (http://www.geodatenzentrum.de/)
Soil texture	Vector	1:250 000	LLUR
Landscape types	Vector	-	LLUR (http://www.umweltdaten.landsh.de)
Water erosion	Vector	1:250 000	LLUR (http://www.umweltdaten.landsh.de)
Wind erosion	Vector	1:250 000	LLUR (http://www.umweltdaten.landsh.de)
Nutrient regulation ES demand—Schleswig-Holstein	Vector	Municipalities	[15]
Nutrient regulation ES demand—Schleswig-Holstein	Vector	See CORINE LULC data set	[15]

In addition to these data sets, the values for the nutrient regulation ES potential published in the ES matrix by Burkhard et al. [10] were adopted.

The ES matrix approach has been developed based upon the assumption that ES are spatially and temporally explicit and therefore can be linked to units in space and time [78]. As stated in the introduction, the ES matrix links individual ES to appropriate geo-biophysical spatial units. The approach allows to asses ES potential, flow and/or demand on a relative scale. Commonly, a relative scale ranging from zero to five is used, representing no relevant ES and very high ES, respectively [2,10,12,13,16,78]. The normalized scale supports the comparability of different services and assessments. In general, the approach allows for the incorporation of different input data. This fact makes the approach convenient for the application in both data-rich and data-scarce study areas. Burkhard et al. [10] applied the ES matrix and assessed several ES potentials and demands based upon qualitative and quantitative data. Some ES potentials were valued basing upon a detailed analysis of the Leipzig-Halle research area of Kroll et al. [79], using regional statistics, thematic maps, regional information and empirical data on flows and demands of the ES [80].

Burkhard et al. [10] selected the CORINE LULC data as the underlying spatial reference data set. The different land cover classes of the data set served as geo-biophysical spatial units. Thus, using different quantification methods and qualitative expert evaluation, which has been based upon experiences from case study ES assessments [79,80], they linked individual ES potential and demand values to each land cover class.

The values range from 0 (no relevant ES potential) to 5 (highest ES potential) and were joined to the CORINE LULC data set using ArcMap 10.3 in order to come up with the corresponding distribution of the nutrient regulation ES potential within the study areas.

The colours of the nodes within the BBN were selected in order to support the understanding of the BBN structure. The nodes of all data sets which were used to predict the nutrient regulation ES potential are blue, with the exception of the grey-coloured node landscape types. The predicted node nutrient regulation ES potential as well as the reclassified nutrient regulation ES potential are greenish in order to visually represent the supply side of the ES, whereas the nutrient regulation ES demand is red in order to represent the opposite. The nutrient regulation ES budget is orange.

## 4 Results

### 4.1 Nutrient regulating ecosystem services in Schleswig-Holstein

**Fig 5:**
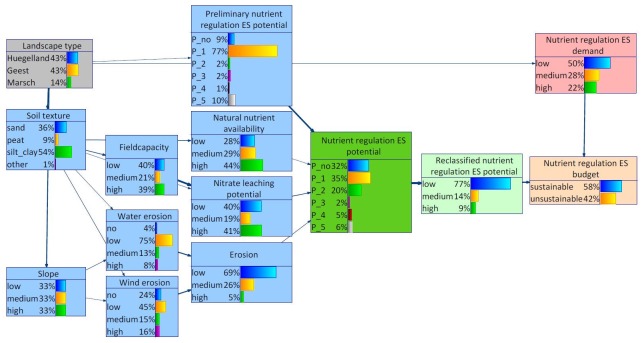
BBN for Schleswig-Holstein. The width of the arcs indicates strength of influence.

From the BBN assessment, the following structure and corresponding probability distributions resulted for Schleswig-Holstein (Fig 5): The states no relevant, low relevant and relevant of the variable nutrient regulation ES potential share a probability of more than 80%, whereby no relevant and low relevant make a contribution of 32% and 35%, respectively. The figure also depicts the overall probability distributions of the nodes depicting further environmental characteristics of the study area as well as the nutrient regulation ES demand and budget nodes. With regard to the nutrient regulation ES budget, the state sustainable is most probable (more than 50%). The width of the arcs distinguishes the influence of the parent node on the corresponding child node. The influence between the landscape types on the preliminary nutrient regulation ES potential node is notably weak. Contrary to that, strong influences can be detected between landscape types and natural circumstances such as soil texture and slope.

#### 4.1.2 Altered distribution

**Fig 6:**
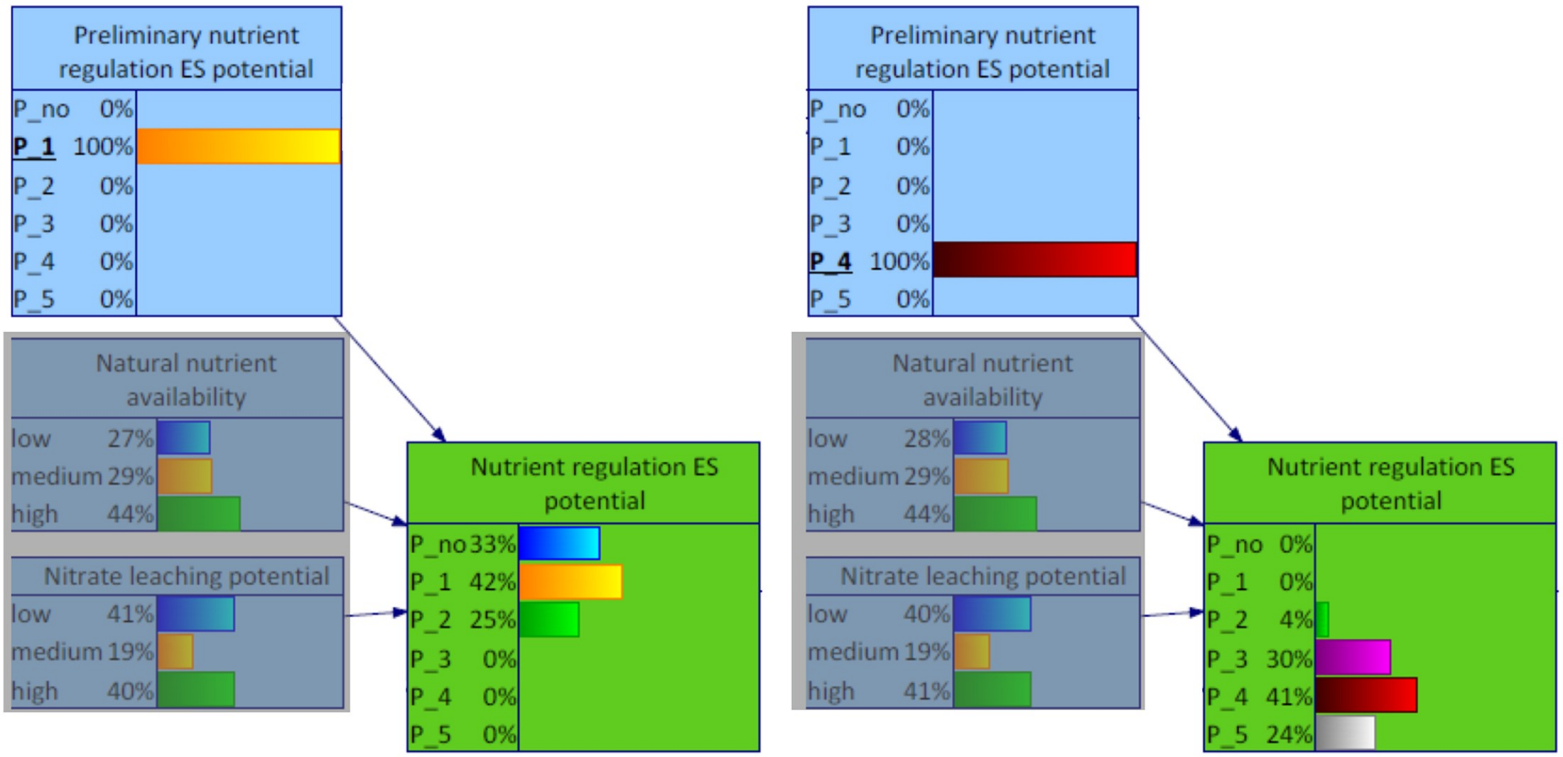
Excerpt of the BBN for Schleswig-Holstein focusing on changing nutrient regulation ES potential level, exemplarily for original low potential (1, left) and high potential (4, right).

Through the integration of additional data into the assessment of the nutrient regulation ES potentials, the probability distribution became diffused. Fig 6 presents this alteration for the former low relevant (left) and high relevant (right) nutrient regulation ES potentials. The shifted probability distributions still peaked at the corresponding former state, but only with around 40 percent. The probability was scattered among neighbouring states.

#### 4.1.3 Regional differentiation

**Fig 7:**
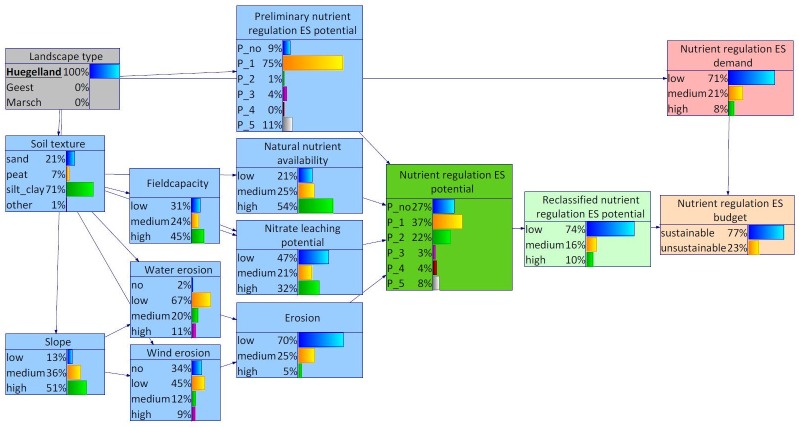
BBN for Schleswig-Holstein, landscape type Hügelland set as evidence.

**Fig 8:**
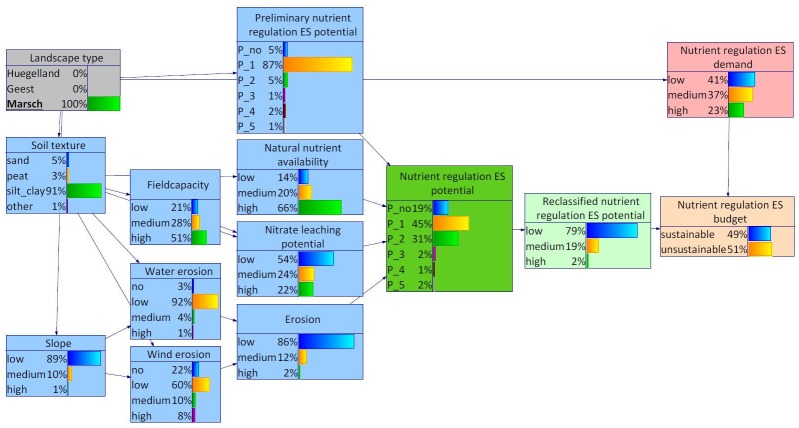
BBN for Schleswig-Holstein, landscape type Marsch set as evidence.

Both, the Hügelland and the Marsch (Figs 7 and 8), were featured with highest probabilities for silty/clayey soils. This distribution was also reflected in the probability distributions of the other site-specific characteristics, in particular natural nutrient availability, field capacity and nitrate leaching potential. As a result, the probability distribution of the nutrient regulation ES potential is highest for low relevant nutrient regulation ES potential. Also the class relevant nutrient regulation ES potential scores a relatively high probability. The probability distribution of the node slope differs most between the Hügelland and the Marsch (Figs 7 and 8). While the Marsch was characterized with lower than average slopes, the opposite was true for the Hügelland. This difference was also reflected in the probability distributions of the child node water erosion.

**Fig 9:**
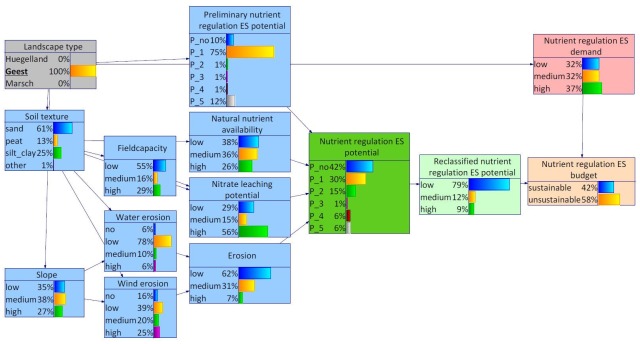
BBN for Schleswig-Holstein, landscape type Geest set as evidence.

For the Geest, the BBN indicated highest probabilities for sandy soils which was in turn also reflected in the probability distributions of the other site-specific characteristics (Fig 9). The probability distributions of the nodes slope and water erosion roughly resembled the average distributions for Schleswig-Holstein. In contrast to that, the probability distribution of the node wind erosion indicated higher wind erosion risks in the Geest than in the rest of the study area. Altogether, the probability distribution of the node (overall) erosion indicated highest risks for erosion in the Geest. Furthermore, the probability distributions indicated generally lower natural nutrient availability and higher nitrate leaching potentials in the Geest compared to the other landscape types. Altogether, this resulted in a probability distribution for the node nutrient regulation ES potential which indicates a lower than average potential nutrient regulation in the Geest area. In addition, the nutrient regulation ES demand in the area was higher than average. With regard to the nutrient regulation ES budget, unsustainability was more probable in the Geest than for the other two landscape types.

### 4.2 Nutrient regulation in the Bornhöved Lakes District

The results from the assessment of the Bornhöved Lakes District scale resemble the outcomes on the scale of the federal state. As described in section 2.1.2, the Bornhöved Lakes District is located at the border of the two landscape types Hügelland and Geest. The Marsch area is not part of the Bornhöved Lakes District which can be recognized in the probability distribution of the corresponding states of the variable landscape types.

**Fig 10:**
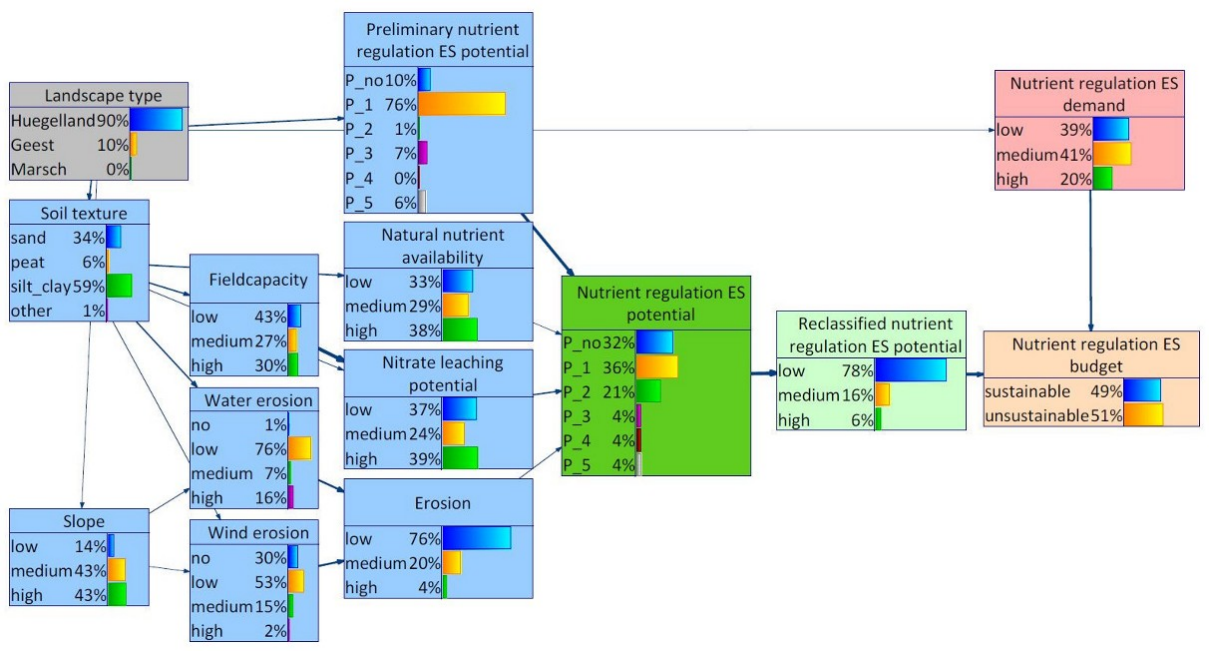
BBN for Bornhöved Lakes District. The width of the arcs indicates strength of influence.

In the Bornhöved Lakes District, the probability distribution for the variable slope differed from the distribution for Schleswig-Holstein (Fig 10). Higher probabilities could be found for the states medium and high. The probability distribution of the nutrient regulation ES demand differed most from the distribution for Schleswig-Holstein. The highest probability was attributed to the state medium instead of low. As consequence, the probability distribution of the nutrient regulation ES budget shifted somewhat resulting in a share of 51% for the state unsustainability (Fig 10).

**Fig 11:**
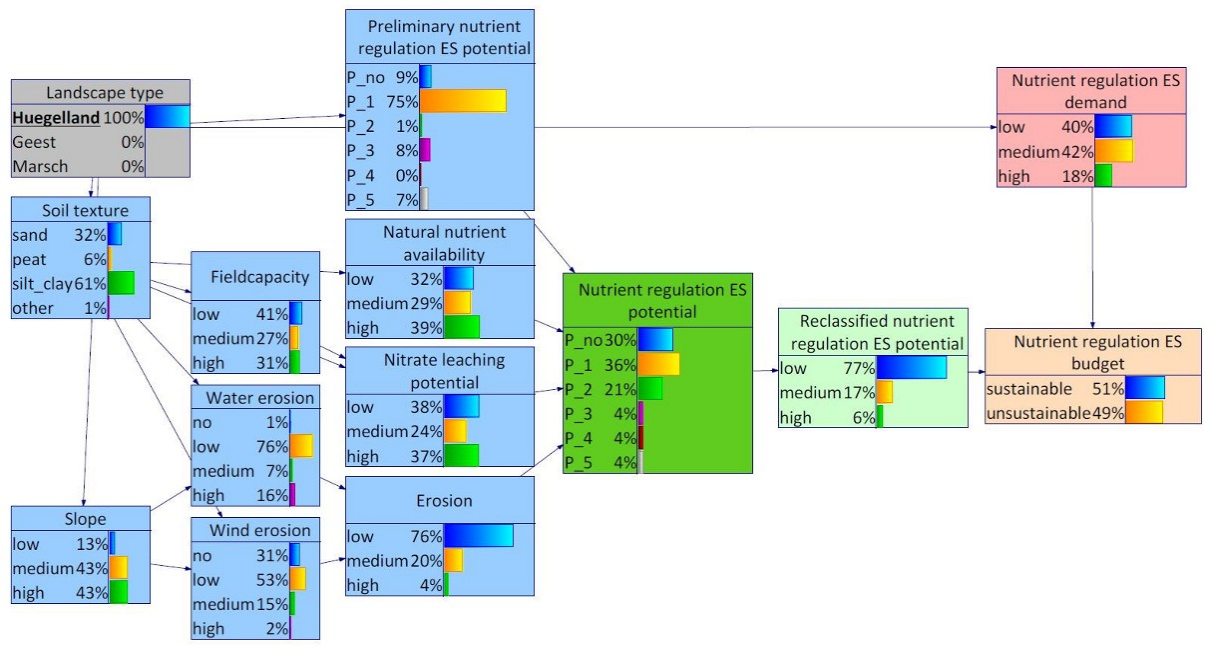
BBN for Bornhöved Lakes District, landscape type Hügelland set as evidence.

**Fig 12:**
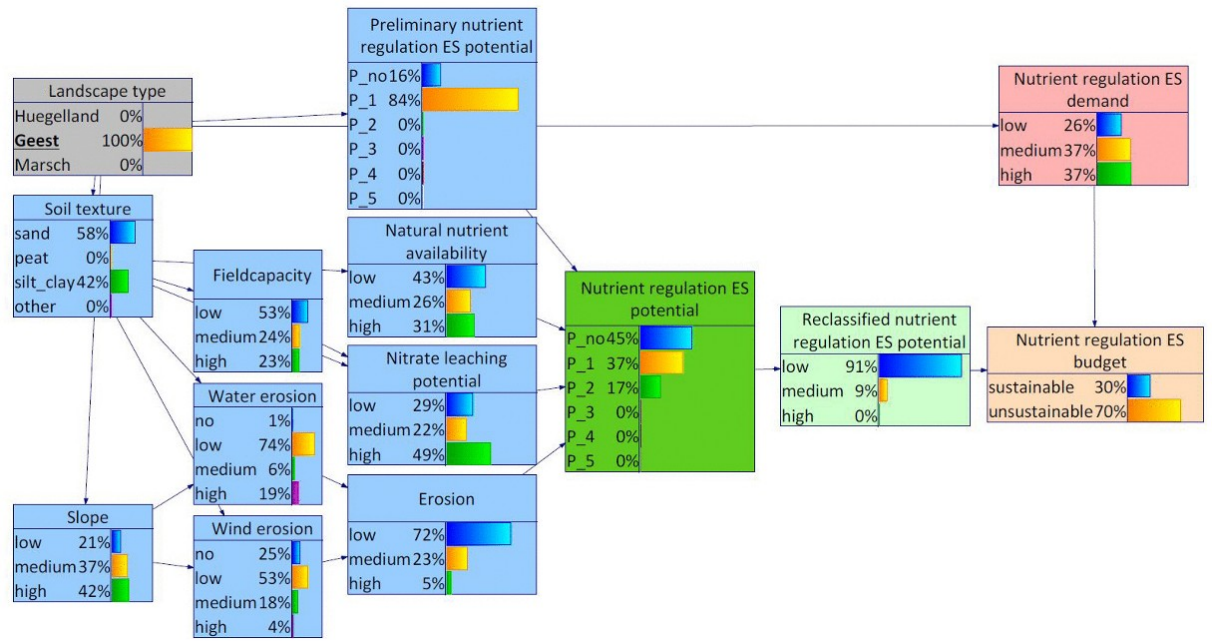
BBN for Bornhöved Lakes District, landscape type Geest set as evidence.

When differentiating between the landscape types Geest and Hügelland within the Bornhöved Lakes District, differences were found with regard to the demand as well as the supply side of the nutrient regulation ES (Figs 11 and 12). Selecting Geest as evidence, the states medium and high both featured probabilities of 37%. In combination with a somewhat lower nutrient regulation ES potential, the state unsustainable of the variable nutrient regulation ES budget reached 70%. The shift on the supply side resulted from many marginal differences in the probability distributions of the environmental conditions, notably soil texture and dependent child variables.

## 5 Discussion

### 5.1 Findings

**Fig 13:**
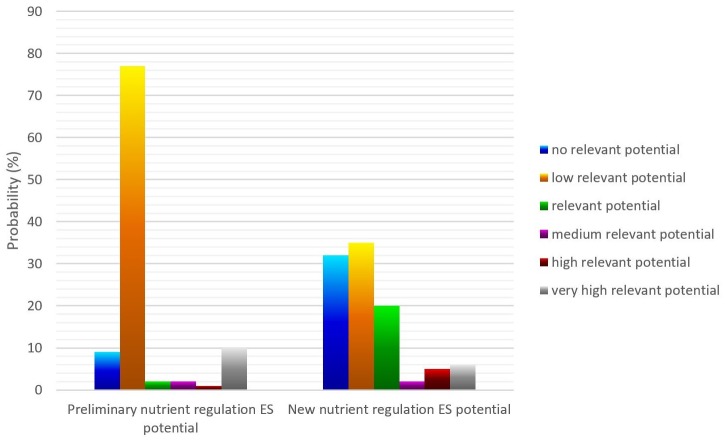
Probability distribution of the preliminary and new nutrient regulation ES potential.

According to the outcomes of the BBN, around one third of Schleswig-Holstein had no relevant nutrient regulation ES potential. More than 50% of the area was characterised by (low) relevant ES potentials. The remaining portion was distributed between medium, high and very high relevant ES potentials. Comparing these values with the original distribution of nutrient regulation ES potential based on the matrix values provided in the ES matrix by Burkhard et al. [10], a broader distribution could be identified (Fig 13). The original distribution peaked more extremely at low relevant ES potentials (with more than 70%). This alteration was also demonstrated for the distribution of low and high relevant nutrient regulation ES potentials separately (Fig 6).

The study revealed a regional differentiation of nutrient regulation ES potentials in Schleswig-Holstein. While the areas of the Hügelland and Marsch obtained higher probabilities for higher ES potentials compared to the whole federal state, the results for the Geest turned out to be considerably lower. The incorporation of the data on site-specific properties was reflected in the outcomes. Regional differences with regard to the probability distributions of the environmental conditions such as slope and soil texture corresponded to the background information of the study areas. For instance, the area of the Geest was characterized by poor, sandy soils (see study area for more information) which are susceptible to wind erosion and nitrate leaching. These soils have relatively low field capacities and natural nutrient availability rates. These circumstances led to the obtained outcomes.

Considering the nutrient regulation ES budget, the results highlighted this regional issue. The Geest obtained high probabilities for an unsustainable nutrient regulation ES budget, as the area is featured by a low potential ES supply and a relatively high ES demand. The Hügelland obtained highest probabilities for a balanced ES budget as the area is featured by an average potential supply and a relatively low nutrient regulation ES demand.

Setting up the BBN for both study areas, the following conclusions can be made with regard to the issue of spatial scales: The time used for setting up the BBN for the study areas did not depend on the size of the study area. While training the BBN nodes, the elapsed time differed for the two different data sets in relation to the amount of data points. This difference can, however, be summed up to minutes only. In our case, different resolution input data had the greatest chance to provoke differences related to the issue of scale. This was the case in our BBN for the node nutrient regulation ES demand. The underlying data sets, which were adopted from Bicking et al. [15], differ in resolution. For Schleswig-Holstein, the demand was specified on the level of municipalities. The data set for the Bornhöved Lakes District was spatially more explicit, based on the polygons of the CORINE LULC shape file. As result, the probability distribution of the variable nutrient regulation ES budget differed considerably from the results on the Schleswig-Holstein scale. This alteration became even more distinct when the BBN for the Bornhöved Lakes District was assessed for the different landscape types. These findings indicate that the analysis on a larger scale and with a lower resolution underestimated the issue.

### 5.2 Strengths vs. limitations and uncertainties

Within this research, we combined quantitative data sets with information from expert evaluation in order to include as much valuable information as possible into the BBN. Based upon our and the experts’ system understanding, we aligned the DAG of the BBN. Thus, the structure of the BBN is solely elicited from experts. However, only four out of the fourteen nodes (the CPTs) have been filled in manually based on expert knowledge. For the remaining ten nodes, the BBN was trained by input data from other sources. Thus, more than 70% of the nodes in the BBN were based upon spatial data sets. However, the target node (nutrient regulation ES potential) can be found under the four nodes with expert-based constructed CPTs. Expert evaluation generally comes along with a range of uncertainties [12,81]. Experts tend to base their evaluations strongly on their personal experience, knowledge, living conditions and attitude [11,12]. As proposed in recent literature [11,82], we aimed to limit the uncertainties with regard to the expert evaluation as we consulted experts from different relevant research fields. However, the number of experts was limited and contributed considerably to the uncertainties of this study. Besides, the expert evaluation was executed by using roundtable discussions. The involved experts stated that this format, which supports knowledge-exchange, enhances the incorporation of an interdisciplinary perspective in the decision making process. Experts were satisfied by the general consent on the cause-effect relationships between the nodes. However, they question the accuracy of the precise probabilities which are inserted into the CPT. Considering the general objective of the study, this uncertainty seems to be tolerable.

We assessed the nutrient regulation ES potential exemplarily for the nutrient nitrogen. As different nutrients vary with regard to chemical as well as physical properties, the processes in the environment differ and thus the structure of the BBN as well as its relations need to be adapted in order to obtain realistic results. Besides, the nodes included in a BBN contain a limited number of states. The data which served as model input were reclassified in order to fit this requirement. Defining different states and/or using a different reclassification scheme alters the distribution of the data, which in turn could result in the delivery of different outcomes with regard to the nutrient regulation ES potential analysis and eventually shift the sustainability decision. One needs to keep in mind that the ES budget node has been included into the network in order to allow for a rough estimation with regard to the balance of the ES demand and supply. That was why the resulting probability distributions should not be over-interpreted but only be regarded as a rough approximation.

The focal objective of the study was to test the application of a BBN as an integrative modelling approach combining the information from the ES matrix with additional data sets. The BBN model did not attempt to compile a complete network resembling complex reality as elaborately as possible. Altogether, we only used a limited number of variables to describe the nutrient regulation ES potential. The complexity of the environmental system was not entirely reflected in the constructed BBN which can be seen as a basic overview. On the one hand, we chose to keep it simple, as it fitted the target of our study. On the other hand, data availability could be identified as major constraint. If an environmental system is to be simulated as much as possible, further site-specific characteristics, such as information on soil organic matter content, landscape structures and precipitation, should be included in the BBN.

According to Hou et al. [81], ecosystem services evaluation on the landscape scale involves various uncertainties. These uncertainties can be associated with issues concerning the initial data and the preferences of respondents (regarding expert evaluation), technical problems, methodological uncertainties and the general complexity of natural systems. All of these issues represent constraints identified in ecosystem service research [81,83] and are valid for the practical implementation of this study. A further constraint arises from the implementation of a rather abstract concept to a specific approach involving particular methodologies and data sets.

## 6 Conclusions

### 6.1 General conclusions

Summing up the outcomes obtained from this study, the hypotheses put forward in the introduction were evaluated:

1. **The inclusion of data on site-specific properties for the assessment of the nutrient regulation ES potential results in a more scattered distribution of the ES potentials compared to the ES matrix values provided by Burkhard et al.** [10]**.**

Yes, the probability distribution for the nutrient regulation ES potential based on the BBN was found to be wider than the original distribution based on the ES matrix values, which evaluated the nutrient regulation ES potentials for CORINE LULC types. The inclusion of more data on site-specific properties resulted in a more diverse pattern in regard to the probabilities of the nutrient regulation ES potentials.

1. **The assessment of the nutrient regulation ES potentials results in a regional differentiation in Schleswig-Holstein.**

Incorporating site-specific properties into the nutrient regulation ES potential assessment revealed the different potentials of the three main landscape types. The probability distribution of the Hügelland and Marsch peaked for low relevant potentials, whereas the Geest was featured with the highest probability for no relevant ES potentials. This regional differentiation became even more obvious concerning the estimated nutrient regulation ES budget (potential supply vs. demand). The area of the Geest unified relatively high probabilities for low nutrient regulation ES potentials and remarkably higher probabilities for high demand for nutrient regulation ES.

1. **The probability distribution of the nutrient regulation ES potential in the Bornhöved Lakes District resembles the distribution for Schleswig-Holstein.**

In general, the two BBNs delivered similar probability distributions. The greatest difference existed between the nutrient regulation ES demand. The Bornhöved Lakes District was characterized by higher demands compared to Schleswig-Holstein. This was reflected in the probability distribution of the nutrient regulation ES budget which identified higher probabilities for an unsustainable ES budget in the Bornhöved Lakes District. Similar to the BBN for Schleswig-Holstein, the BBN for the Bornhöved Lakes District also identified the Geest as an area with high probabilities for relatively low nutrient regulation ES potentials and relatively high nutrient regulation ES demand. However, minor differences became apparent with regard to the magnitude of the mismatch. The mismatch at the scale of the Bornhöved Lakes District depicted an even clearer picture.

It is striking that in spite of the distinct spatially varying distribution of the environmental conditions, land management with regard to agricultural practices has not been adapted to these circumstances. Land management aiming to adjust practices in accordance with the regional environmental conditions would result in more sustainable agriculture. Such a site-specific agriculture would help to save resources while at the same time safeguard the environmental conditions and biodiversity of our ecosystems.

In summary, it can be stated that the BBN is an appropriate method in order to integrate additional data to the spreadsheet matrix approach for assessing ecosystem services. The possibility to include both qualitative and quantitative data to the network emphasizes the convenience of the approach. After the execution of this study, we share the belief that the use of BBNs to model ecosystem services using both empirical data and expert knowledge is promising [22,68].

This fact increases the value of the BBN for ES research as data from the different research domains (environmental, socio-cultural and economic) can easily be integrated. The added value of this BBN approach compared to the original ES matrix assessment by Burkhard et al. [10] is the consideration of environmental data other than CORINE land use/land cover. The influence of several natural conditions on the nutrient regulation ES potential has been incorporated. In our case, the integration of further spatially explicit data resulted in a distinct regional pattern with regard to the ES nutrient regulation.

Outcomes which are based on higher resolution input data emphasised the unsustainable situation with regard to agricultural practices.

### 6.2 Future research

If data on cultivated crop types and their properties in the study areas would be incorporated into the BBN, more elaborate conclusions could be drawn on sustainability with regard to the nutrient regulation ES potential. In this context, the integration of land management options into the BBN would be an interesting approach. Land management options such as tillage are crucial for the determination of the nutrient regulation ES potential of agricultural grounds. The timing as well as the tillage technique employed influence the potential for soil loss through erosion and simultaneously the potential for nutrient loss in the system. Especially in combination with scenario assessments, this integration could deliver highly interesting results. In the future, it would be interesting to include further ES into the BBN. We find it exciting to assess whether one can come up with one pervasive network which could be applied for ES assessments on top of the matrix approach by Burkhard et al. [10].

## Supporting information

S1 Table Processed data BBN—Schleswig-Holstein. (CSV)

S2 Table Processed data BBN—Bornhöved Lakes District. (CSV)
